# Determinants of active pulmonary tuberculosis in Ambo Hospital, West Ethiopia

**DOI:** 10.4102/phcfm.v7i1.608

**Published:** 2015-09-07

**Authors:** Tenna Ephrem, Bezatu Mengiste, Frehiwot Mesfin, Wanzahun Godana

**Affiliations:** 1 Oromia Regional Health Bureau, Addis Ababa, Ethiopia; 2 College of Health and Medical Sciences, Haramaya University, Ethiopia; 3 College of Medicine and Health Sciences, Arba Minch University, Ethiopia

## Abstract

**Objectives:**

The aim of this study was to determine factors associated with active pulmonary tuberculosis seen in cases in Ambo Hospital, Ethiopia.

**Design:**

A facility-based prospective case-control study.

**Setting:**

Patients attending Ambo Hospital from 01 December 2011 to 29 March 2012.

**Participants:**

The sample included 312 adult patients attending Ambo Hospital. The main outcome measure was presence of active pulmonary tuberculosis (TB).

**Explanatory measures:**

Age, gender, occupation, educational status, marital status, place of residence, patient history of TB, family history of TB, human immunodeficiency virus (HIV) infection, smoking, alcohol intake, khat chewing, body mass index (BMI), employment, diabetes, history of asthma, previous history of worm infestation, history of hospitalisation, number of adults living in the household (HH), person per room, housing condition.

**Results:**

A total of 312 study participants, including 104 active pulmonary tuberculosis (PTB) cases (cases) and 208 non-active PTB cases (controls), were recruited for the present study. Having one or more family member with a history of TB (OR = 4.4; 95% CI: 1.50–12.90), marital status (OR = 7.6; 95% CI: 2.2–12.6), male gender (OR = 3.2; 95% CI: 1.4–7), rural residence (OR = 3.3; *P* = 0.012), being a current or past smoker (OR = 2.8; 95% CI: 1.1–7.2), BMI < 18.5 (OR = 2.1; 95% CI: 1.03–4.2), HIV infection (OR = 8.8; 95% CI: 2.4–23.8) and a history of worm infestation (OR = 6.4; 95% CI: 2.6–15.4) remained significant independent host-related factors for active PTB.

**Conclusion:**

Patients who came from a compound with more than two HHs were more likely to develop active PTB than those who came from a compound with only one HH. Those who lived in houses with no windows were more likely to develop active PTB than those who lived in houses with one or more windows, had a family history of TB, lived in rural areas. Sex of the patient was a predicting factor. Not being the owner of the house was significantly more associated with active PTB. Measures taken to reduce the prevalence and burden of active PTB should consider these determinant factors.

## Abstract (fr)

**Les déterminants de tuberculose pulmonaire active à l’hôpital d’Ambo, en Ethiopie occidentale.**

**Objectifs:**

Le but de cette étude était de déterminer les Facteurs associés de Tuberculose pulmonaire active à l’Hôpital d’Ambo.

**Conception:**

Une étude cas-témoins prospective au sein d’un établissement.

**Cadre:**

les patients qui sont venus à l’hôpital d’Ambo du 15 décembre 2011 au 29 mars 2012.

**Participants:**

312 patients adultes sont venus à l’hôpital d’Ambo. Résultats principaux: présence de Tuberculose pulmonaire active.

**Mesures explicatives:**

Age, Sexe, Profession, niveau d’études, état civil, lieu de résidence, état tuberculeux du patient, antécédents de Tuberculose, infection VIH, tabagisme, consommation d’alcool, mastication de khât, BMI, emploi, diabète, antécédents d’asthme, antécédents d’infection par des vers, hospitalisations passées, nombre d’adultes vivant au HH, nombre de personnes par chambre, conditions de logement.

**Résultats:**

On a recruté 312 participants pour cette étude, 104 cas de Tuberculose pulmonaire active (PTB) et 208 cas de PTB non active (Contrôles). Les patients ayant un membre ou plus de leur famille atteint de Tuberculose (OR = 4.4, 95% CI : 1.50−12.90), les célibataires (OR 7.6, 95% CI : 2.2−12.6), les personnes de sexe masculin (OR 3.2, 95% CI : 1.4–7), les personnes des régions rurales (OR 3.3, *p =* 0.012), les fumeurs ou anciens fumeurs (OR 2.8, 95% CI : 1.1−7.2), la sous-alimentation (BMI < 18.5) (OR 2.1 et 95% CI : 1.03−4.2), l’Infection VIH (OR 8.8 et 95% CI : de 2.4−23.8), les antécédents d’infection de vers (OR 6.4 et 95% CI : 2.6−15.4) sont indépendamment des facteurs importants propres aux personnes atteintes de Tuberculose pulmonaire active.

**Conclusions:**

les patients provenant d’un composé de plus de deux HH étaient plus susceptibles de développer la Tuberculose pulmonaire active que ceux qui provenaient d’un composé avec un seul HH. Ceux qui vivent dans des maisons sans fenêtres étaient plus susceptibles de développer la Tuberculose pulmonaire active que ceux qui vivent dans des maisons à une fenêtre ou plus. Ceux qui n’étaient pas propriétaire de la maison étaient beaucoup plus susceptibles d’être atteint de Tuberculose pulmonaire active. Les mesures prises pour diminuer la prévalence et le fardeau de la Tuberculose pulmonaire active devraient considérer ces facteurs décisifs.

## Introduction

Tuberculosis (TB) is an airborne bacterial disease. The causal agent is the tubercle bacillus *Mycobacterium tuberculosis*, and sometimes *Mycobacterium bovis* or *Mycobacterium africanum*.^1,2^ The most common form of disease, caused by *M. tuberculosis,* is pulmonary tuberculosis (PTB). In the lungs, the bacterium is taken up and if it is not contained by the immune system, it is able to grow uncontrollably, resulting in the subsequent development of TB. The pulmonary form of TB is infectious because it is transmitted through aerosol whenever individuals with active PTB cough, sneeze, talk or laugh, and droplets in the air are inhaled by those who are in close contact with the infectious case.^3,4,5,6^

Globally, TB represents one third of the world's health problems; roughly two billion people are affected.^7,8^ Annually, an estimated eight to ten million people develop TB owing to primary infection, reactivation or re-infection.^8^ In 2009, there were an estimated 9.4 million new cases, 14 million existing cases and 1.68 million deaths from TB. Most cases were in the South-East Asia, African and Western Pacific regions (35%, 30% and 20%, respectively).^7,9^ Ninety-five per cent of TB cases in low-income countries are amongst people between 15 and 50 years old.^10,11^

TB is still the leading cause of infection in some tropical countries. In some countries, overcrowding in urban slums further aggravates the situation. The co-existence of human immunodeficiency virus (HIV) infection and TB worsen the health of the population.^4,8,12^

Ethiopia is amongst the world's 22 high-burden TB countries. An overall incidence rate (people acquiring PTB) increases from time to time. The trend of increment in incidence rate rose from 331 per 100 000 in 2000 to 378 per 100 000 in 2007.^1,13^

TB is a multifactorial disease in which environmental and individual factors contribute to the disease process.^5,14^ An individual with acquired immune deficiency syndrome (AIDS) is 110–170 times more likely to develop TB than a person with no known risk factors, and an individual with HIV has a risk of 50–110 times greater than a person with no known risk factors.^3^ Individuals with HIV have deficient immune systems for immunologic containment. Globally, TB is the leading cause of mortality of people with HIV.^15^

Many factors are related to TB, such as smoking,^16,17^ and rates of TB infection, disease and mortality are significantly higher amongst smokers.^18,19,20^ Another factor that is related to TB is diabetes mellitus (DM)^2,13^ and a recent meta-analysis found that patients with DM were three times more likely to have TB than controls.^21^ Drinking alcohol excessively also increases the likelihood of developing TB by three times more than in those who do not. Generally, there are different explanations for this scenario.^22^

Relatively little data are available concerning the association between nutrition or low body mass index (BMI) and TB. One recent review on TB and low BMI indicated a strong dose–response relationship with TB incidence; in the six summarised studies, TB increased exponentially as BMI decreased.^23^ Owing to the widespread global prevalence of under-nutrition, on a population level, the effect of this risk factor has been predicted to be substantial.^24^ Deficiencies for protein and micronutrients such as vitamin D, arginine and zinc^3^ increase the risk of TB. There is a documented inverse relationship between BMI and risk of progression from TB infection to TB disease.^25^

Concerning individual host factors, results from multivariate analyses from three West African countries (Guinea, Guinea-Bissau and The Gambia) identified TB to be related to male gender, a family history of TB, absence of a Bacillus Calmette–Guérin (BCG) scar, smoking, alcohol use, anaemia, seropositive HIV status, and history of and treatment for worm infestation.^14^

A study conducted in Gonder, Ethiopia on TB risk factors for PTB found that there is a strong relationship between TB and HIV status (7.8 times higher in cases) and between TB and intestinal helminth infestation (4.2 times higher in cases).^26^

TB burden follows a socio-economic gradient; therefore, poverty and its related conditions are strong social and environmental determinants of TB.^24^ Studies from Russia, The Gambia and south-west Ethiopia revealed that family history of TB was independently related to active PTB.^26,27,28^ Furthermore, there was some evidence that this effect was higher when the previous or former TB case was in close family, as compared with unrelated household members. The clustering of TB within families could reflect not only the place of transmission within the family, but also a genetic contribution to the susceptibility to active PTB.^29^

Efforts to decrease the rates of TB must focus on controlling transmission and identifying associated factors as a means of reducing the overall burden of the disease. It has been observed that not all patients who have PTB transmit the disease; therefore, research must include an examination of specific determinant factors to show which influence and result in development of the disease without difference in the infection status. The present research focused on PTB patients to identify the factors related to developing active PTB in comparison with people without TB.

## Research methods and design

### Study area and period

The study was conducted in Ambo Hospital, the zonal referral hospital of the West Shoa zone of Oromia Regional State, from 15 December 2011 to 29 March 2012. The town Ambo is located 115 km away from Addis Ababa in the western part of Ethiopia. It is the only governmental institution in the zone that has a functional X-ray machine for chest radiography to detect smear-negative TB cases. The hospital serves the population of the zone and neighbouring districts as a referral hospital.

### Study design

A facility-based prospective case-control study was conducted amongst patients visiting the adult outpatient department (OPD) of Ambo Hospital.

Active PTB patients (smear-positive and smear-negative TB cases) diagnosed in the OPD of the hospital during the study period were included in the study. Active PTB was defined as bacteriologically, radiologically or clinically detected TB. A smear-positive or smear-negative TB case was defined as one for which chest radiography findings were consistent with TB, there was a lack of response to a trial of broad-spectrum antimicrobial agents (excluding anti-TB drugs and fluoroquinolones) and a physician's judgement on detecting a case as TB. The TB screening and diagnosis protocol of the World Health Organization (WHO) was used.

Controls were OPD patients who were free of active PTB and TB screening signs and symptoms during the study period. According to the WHO standards, individuals who are free of these constitutional symptoms are declared free of active TB although they may be infected with the TB bacterium.

### Study population

The study population included all patients aged 15 years and older who attended the OPD of Ambo Hospital during the study period. On average, there were 73 OPD patients per day.

### Sample size

Sample size was estimated using the Open-Epi sample size calculator software (CDC, Atlanta) according to the following factors:

- power: 80%
- confidence interval (CI): 95%
- sample ratio of controls to cases: 2:1
- proportion of family history of PTB amongst controls: 17%
- odds ratio (OR) of family history of TB from previous studies^14^ (in three West African countries): 2.38

With the Fleiss continuity correction, the sample size was set at 95 cases and 189 controls, and with a 10% non-respondent rate the sample size became 104 cases and 208 controls. The total sample size was therefore 312.

### Sampling procedure

All active PTB patients (smear-positive and smear-negative TB cases) aged 15 years and older at the OPD of the hospital during the study period were included. The first OPD attendee who was free of active PTB and aged 15 years or older following each case was approached to serve as a control in the study. If these individuals refused participation, the next eligible OPD attendee was approached.

### Study design

The outcome variable of the study was active PTB status. Independent variables included the following host-related variables: age, gender, occupation, educational status, marital status, place of residence, patient history of TB, family history of TB, HIV status, smoking history, alcohol intake, khat chewing, BMI, employment, diabetes status, history of asthma, previous history of worm infestation, history of hospitalisation, and history of incarceration. Environment-related variables were as follows: number of households (HHs) in the compound, number of adults in the HH, number of people per room, wall type, floor type, type of roof and/or ceiling, number of windows, availability of electricity, separate kitchen, latrine, ownership of the house, and type of cooking fuel.

### Data collection

A pre-tested structured questionnaire was used to collect all data on the study variables, a beam balance scale was used to measure weight, and a tape measure to measure the height of the respondents. All OPD patients were interviewed by data collectors for their cases at exit to identify PTB cases and consult with the OPD physician to confirm the case. Study participants answered a structured questionnaire that was conducted in their own language by trained diploma nurses. Information was collected on a wide range of potential host-related and environment-related risk factors for active PTB. Host information included basic demographic data (age, gender and ethnicity), past medical history of asthma and diabetes, history of smoking and alcohol consumption, schooling and category of occupation, and previous history of TB. The environmental factors included the type of cooking fuel used, building structure and materials of the house, housing conditions, whether there was another member of the HH with TB disease, and a crowding index (such as number of adults living in the HH, number of HHs in the compound and people per room). All HIV test results of the cases and controls were recorded, the height and weight of the respondents were measured, and BMI was calculated.

### Data analysis

The data were entered into a pre-drafted coding sheet on Epi Info software (version 3.5.3) by two different data clerks. A binary logistic analysis with conditional method was used to measure the association between the dependent variable and independent variables using an OR and 95% CI. Statistical significance was set at α ≤ 0.05. In an attempt to identify the relative effects of explanatory variables on the outcome variable, hierarchical multivariate analyses were applied. Independent predictor variables with a *P*-value < 0.25 were entered into the final regression model, based on the likelihood ratio for further analyses in different models.

A backward stepwise entry method was used for the logistic regression for each model. Finally, a combined multivariate model was constructed from the individual host- and environment-related risk factors for analysis.

### Data quality control

A properly designed and pre-tested questionnaire was used. Training, close supervision during data collection and double data entry were implemented as quality control measures.

### Ethical considerations

Ethical approval and clearance for this study were obtained from the Institutional Research Ethics Review Committee of the College of Health and Medical Sciences, Haramaya University. At all levels, officials were contacted and permission from administrators was secured. All the necessary explanation about the purpose of the study and its procedures was explained with the assurance of confidentiality. Written and verbal consent from the study participants were also secured.

## Result and discussion

### Results

A total of 312 study participants (104 active PTB cases [cases] and 208 non-active PTB cases [controls]) were recruited. The median age of cases and controls was 32 and 30, respectively. The majority of the study participants (93.9%) were of the Oromo ethnic group. More than half (58.3%) of the respondents were orthodox in their religion (Table 1).

**TABLE 1:** Demographic characteristics of pulmonary tuberculosis cases and controls in Ambo Hospital, Western Ethiopia, 2011 and 2012.

Variables	Cases *n* (%)	Controls *n* (%)	Total *n* (%)
**Age**			
Mean(standard deviation)	35 (13.4)	35.7(16.1)	35.5 (15.1)
Median (Range)	32 (64)	30 (58)	31 (66)
**Religion** (%)			
Orthodox	66 (63.5)	116 (55.8)	182 (58.3)
Protestant	33 (31.7)	83 (39.8)	116 (37.2)
Muslim	3 (2.9)	7 (3.4)	10 (3.2)
Other	2 (1.9)	2 (1.0)	4 (1.3)
**Ethnicity **(%)			
Oromo	94 (90.4)	199 (95.7)	293 (93.9)
Amhara	5 (4.8)	8 (3.8)	13 (4.2)
Other	5 (4.8)	1 (0.5)	6 (1.9)

### Host-related factors

There were more male patients amongst the cases (74; 71.2%) than amongst the controls (94; 45.2%). The majority of the cases (64; 61.5%) and controls (141; 67.8%) were married; more widowed or divorced patients were part of the cases group (14; 13.5%) than the control group (9; 4.3%). A total of 66 case patients (63.5%) and 109 of the controls (52.4%) were from rural areas. Patient history of TB was higher amongst cases (15; 14.4%) than amongst controls (12; 5.8%). Family history of TB was also higher in cases (20; 19.2%) than in controls (12; 5.8%). Current or past history of smoking was more common amongst cases (24; 23.1%) than amongst controls (15; 7.2%). Khat was more commonly chewed in cases (13; 12.5%) than in controls (11; 5.3%). Under-nutrition (BMI < 18.5) was higher amongst cases (66; 63.5%) than amongst controls (87; 41.8%). HIV infection was higher in cases (21; 20.2%) than in controls (6; 2.9%) (Table 2).

**TABLE 2:** Bivariate analysis of host-related factors associated with active pulmonary tuberculosis in Ambo Hospital, Western Ethiopia, 2011 and 2012.

Variables	Cases (%)	Controls (%)	COR (95% CI)	*P*-value
**Age**†				
15–24	28 (26.9)	56 (26.9)	1.00	
25–34	22 (21.2)	64 (30.8)	0.68 (0.35, 1.34)	0.27
35–44	25 (24)	43 (20.7)	1.16 (0.60, 2.27)	0.66
> 44	29 (27.9)	45 (21.6)	1.29 (0.67, 2.47)	0.45
**Gender**†				
Female	30 (28.8)	114 (54.8)	1.00	
Male‡	74 (71.2)	94 (45.2)	3.0 (1.8, 5.0)	0.000
**Educational status**				
No formal education	42 (40.4)	90 (43.3)	0.89 (0.55, 1.43)	0.63
Formal education	62 (59.6)	118 (65.7)	1.00	
**Marital status**†				
Married	64 (61.5)	141 (67.8)	1.00	
Single	26 (25)	58 (27.9)	1.0 (0.57, 1.7)	0.988
Widowed/divorced‡	14 (13.5)	9 (4.3)	3.4 (1.4, 8.3)	0.007
**Residence**†				
Urban	38 (36.8)	99 (46.7)	1.00	
Rural	66 (63.5)	109 (52.4)	1.6 (0.97, 2.56)	0.064
**Occupation**†				
Civil/public servant	17 (16.3)	46 (22.1)	1.00	
Farmer	45 (43.3)	87 (41.8)	1.4 (0.72, 2.7 )	0.32
Merchant	9 (8.7)	12 (5.8)	2.0 (0.73, 5.7)	0.18
Student	12 (11.5)	43 (20.7)	0.76 (0.32, 1.76)	0.52
Daily labour‡	14 (13.5)	11 (5.3)	3.4 (1.3, 9.)	0.012
Other	7 (6.7)	9 (4.3)	2.1 (0.77, 6.54)	0.20
**Patient history of TB**†				
No	89 (85.6)	169 (94.2)	1.00	-
Yes‡	15 (14.4)	12 (5.8)	2.8 (1.24, 6.12)	0.013
**Family history of TB**†				
No	84 (80.8)	196 (94.2)	1.00	-
Yes‡	20 (19.2)	12 (5.8)	3.9.0 (1.82, 8.3)	0.000
**Smoking**†				
Never	80 (76.9)	193 (92.8)	1.00	-
Current/past‡	24 (23.1)	15 (7.2)	3.9 (1.9, 7.6)	0.000
**Khat chewing**†				
Never	91 (87.5)	197 (94.7)	1.00	-
Current/past‡	13 (12.5)	11 (5.3)	2.6(1.1, 5.9)	0.029
**BMI**†				
< 18.5‡	66 (63.5)	87 (41.8)	2.4 (1.5, 3.9)	0.000
≥ 18.5	41 (36.5)	121 (58.2)	1.00	-
**Employment**				
Employed	22 (21.2)	48 (23.1)	1.00	-
Unemployed	82 (78.8)	160 (78.8)	1.12 (0.63, 2.0)	0.70
**HIV status**‡				
Negative	83 (79.8)	202 (97.1)	1.00	-
Positive‡	21 (20.2)	6 (2.9)	8.5 (3.32, 21.9)	0.000
**History of worm infestation**‡				
No	65 (62.5)	185 (88.9)	1.00	-
Yes‡	39 (37.5)	23 (11.1)	4.8 (2.7, 8.7)	0.000
**Incarceration in last 2 years**				
No	98 (94.2)	201 (96.6)	1.00	-
Yes	6 (5.8)	7 (3.4)	1.8 (0.58, 5.37)	0.32

†*,* Variables with at least one category significant at 25% for further analysis; *‡,* Significant at 5%.

BMI, body mass index; COR, crude odd ratio; HIV, human immunodeficiency virus.

In the multivariate analysis, male patients were 3.2 times more likely (95% CI: 1.4−7.0) to develop active PTB than female patients. Patients who were from rural areas were 3.3 times more likely to develop active PTB than those who were from urban areas (*P* = 0.012). Family history of TB had a significant association with active PTB (Table 2). Under-nutrition (BMI < 18.5) was significantly associated with the development of active PTB, with an adjusted OR of 2.1 and 95% CI of 1.03–4.2. HIV infection was found to have a high association with active PTB, with an adjusted OR of 8.8 and 95% CI of 2.4–23.8. A history of worm infestation was also significantly associated with active PTB, with an adjusted OR of 6.4 and 95% CI of 2.6–15.4 (Table 3).

**TABLE 3:** Environmental factors related to active pulmonary tuberculosis in Ambo Hospital, Western Ethiopia, 2011 and 2012.

Variables	Cases (%)	Controls (%)	Cr OR (95% CI)	Cr *P*-value
**HHs in the compound**†				
1	67 (64.4)	168 (80.8)	1.00	-
> 1	37 (35.6)	40 (19.2)	2.32 (1.4, 4)	0.002
**Adults living in the HH**				
1–5	63 (61.8)	120 (58.5)	1.00	-
> 5	39 (38.2)	85 (41.5)	0.9 (0.54, 1.4)	0.56
**Persons per room**†				
≤ 2	23 (22.1)	64 (30.8)	1.00	-
> 2	81 (77.9)	144 (69.2)	1.57 (0.9, 2.7)	0.104
**Wall type**				
Cement	7 (6.7)	17 (8.2)	1.00	-
Mud	97 (93.3)	191 (91.8)	1.2 (0.5, 3.1)	0.65
**Floor type**†				
Cement	9 (8.7)	36 (17.3)	1.00	-
Earth/other	95 (91.3)	172 (82.7)	2.2 (1.0, 4.8)	0.044
**Roof type**†				
Corrugated iron sheet	83 (79.8)	188 (90.4)	1.00	-
Thatch	21 (20.2)	20 (9.6)	2.4 (1.2, 4.6)	0.011
**Ceiling**				
Yes	50 (48.1)	93 (44.7)	1.00	-
No	54 (51.9)	115 (55.3)	0.87 (0.55, 1.4)	0.57
**Windows**†				
Present	79 (76)	188 (90.4)	1.00	-
Absent	25 (24)	20 (9.6)	3.0 (1.56, 5.66)	0.001
**Electricity**				
Yes	54 (51.9)	98 (47.1)	1.00	-
No	50 (48.1)	110(52.9)	0.83 (0.83, 1.3)	0.42
**Electric cooker**				
Present	10 (6.9)	14 (6.7)	1.00	-
Absent	94 (90.4)	194 (93.3)	0.68 (0.3, 1.6)	0.37
**Separate kitchen**				
Yes	82 (78.8)	154 (74)	1.00	-
No	22 (21.2)	54 (26.0)	0.77 (0.44, 1.3)	0.35
**Ownership of house**†				
Yes	66 (63.5)	180 (86.5)	1.00	-
No	38 (36.5)	28 (13.5)	3.7 (2.1, 6.5)	0.000

†, Significant at 25% level of significance.

CI, confidence interval; Cr, crude; HH, household

### Environment-related factors

In the bivariate analysis of environmental factors for active PTB, the number of HHs in the compound, number of persons per room, type of house floor, type of house roof, number of windows and ownership of the house were all found to be associated with active PTB. Of the 12 variables entered into the bivariate analysis, only six predictor variables were significantly associated with active PTB cases (see Table 3).These variables were included for further analyses in multivariate models.

In the combined multivariate analysis of host- and environment-related factors, patients who lived in houses with more than one HH in the compound were significantly more likely to develop active PTB than with those who lived in HHs with only one house in the compound (adjusted OR: 2.2; 95% CI: 1.02–4.8). Patients who lived in houses with no windows were more likely to develop active PTB than those who lived in houses with one or more windows (OR: 4.3; 95% CI: 1.7–11.0). Not being the owner of the house was found to have an association with active PTB, with an adjusted OR of 10.8 and 95% CI of 3.85–13.3 (Table 4).

**TABLE 4:** Combined environmental- and host-related factors for active pulmonary tuberculosis: multivariate analysis (*n* = 312) in Ambo Hospital, Western Ethiopia, 2011 and 2012.

Variables	Cases	Controls	Adj OR (95% CI)	Adj *P*-value
**Gender**				
Female	30	114	1.00	-
Male	74	94	3.16 (1.4, 7.0)	0.005
**Marital status**				
Married	64	141	1.00	-
Single	26	58	7.6 (2.2, 12.6)	0.001
Widowed/divorced	14	9	3.3 (0.7, 8.5)	0.12
**Residence**				
Urban	38	99	1.00	-
Rural	66	109	3.3 (1.3, 8.6)	0.012
**Patient history of TB**				
No	89	169	1.00	-
Yes	15	12	2.7 (0.8, 8.7)	0.09
**Family history of TB**				
No	84	196	1.00	-
Yes	20	12	4.4 (1.5, 12.9)	0.008
**Smoking**				
Never	86	193	1.00	-
Current/past	18	15	2.8 (1.1, 7.2)	0.034
**BMI**				
< 18.5	66	87	2.1 (1.03, 4.2)	0.042
≥ 18.5	38	121	1.00	-
**HIV status**				
Negative	83	202	1.00	-
Positive	21	6	8.8(2.4, 23.8)	0.001
**History of worm infestation**				
No	65	185	1.00	-
Yes	39	23	6.4 (2.6, 15.4)	0.001
**No of HHs in the compound**				
1	67	168	1.00	-
> 1	37	40	2.2(1.02, 4.8)	0.046
**Persons per room**				
≤ 2	23	64	1.00	-
> 2	81	144	4.1 (1.7, 9.7)	0.001
**Floor type**				
Cement	9	36	1.00	-
Earth/other	95	172	4.5 (1.3, 15.7)	0.018
**Window**				
Present	79	188	1.00	-
Absent	25	20	4.3 (1.7, 11.0)	0.003
**Ownership of house**				
Yes	66	180	1.00	-
No	38	28	10.8 (3.85, 13.3)	0.001

Adj, adjusted; BMI, body mass index; HH, household; OR, odds ratio.

## Discussion

In the present study, the risk factors for active PTB amongst patients at Ambo Hospital were as follows: being male, being single, living in a rural area, having a history of household exposure to a known TB case, smoking, a BMI < 18, HIV infection and a history of worm infestation. These were independent predictors of active PTB. It was also found that having more than one HH in the compound, more than two people living per room, an absence of windows and not being the owner of the house were factors independently associated with active PTB.

Cigarette smoking was independently associated with active PTB, with a clear effect from duration of smoking. This finding is in line with previous studies elsewhere (West African countries, Spain and India), in which a similar association between tobacco smoking and PTB was reported.^14,29,30^ Similarly, several systematic reviews have found that rates of TB infection, disease and mortality are significantly higher amongst smokers.^18,19,20^ It is reported that smoking results in histological changes in the lower respiratory tract, including bronchial inflammation, fibrosis, vascular intimal thickening and destruction of alveoli.^16^ These changes affect epithelial function (e.g. reduced ciliary activity, difficulty in clearance of inhaled substances and altered vascular and epithelial permeability). The results of the current study therefore supports the hypothesis that there is an increased risk of smokers developing active PTB, which is commonly attributed to physiological changes in the lungs caused by long-time smoking.^16,30^ As smoking is becoming an increasing public health problem in low-income countries, especially amongst younger age groups, these results reinforce the need for appropriate public health interventions, as recommended by the WHO Framework Convention on Tobacco Control, which is aimed at helping younger individuals not to develop active PTB.^31^ This issue can be adequately addressed in TB control programme policies. In the meantime, TB-control personnel should have counselling skills to advise TB-infected individuals and their relatives about the advantages of stopping smoking.

HIV infection is amongst the determinants that have measurably increased the risk for active TB and has had a substantial effect on altering TB incidence rates over the past few decades. At a population level, rising HIV prevalence is strongly associated with dramatic increases in PTB notification rates. This is especially so in sub-Saharan Africa, where there are high HIV infection and prevalence rates, and new TB infections are three to five times higher than in 1980.^2^

In the present study, HIV infection was a significant independent risk factor for active PTB, which is consistent with previous studies conducted in African countries.^14^ According to the WHO, 30% of TB cases amongst 15- to 49-year-old adults in sub-Saharan Africa are commonly because of HIV,^13^ and every year nearly a 10% continuous increase in TB is expected in countries with high HIV infection rates. The major factors contributing to the increased number of HIV-associated TB cases in Africa are the increased risk of reactivation of latent TB infection in HIV-infected persons because of decreased immunity, and the increased risk of progression of TB disease because of HIV infection, which will, in turn, result in increased TB transmission rates in the community.

The present study showed that under-nutrition (BMI < 18.5) was an independent determinant factor for active PTB, which is in agreement with previous studies.^24,26^ There are relatively little data on the relationship between BMI and TB, which may be due to the complexity of mechanisms linking nutrition, the immune system and TB, and the difficulties in identifying the relevant biologic markers. One recent review on TB and low BMI indicated that a strong dose–response relationship with TB incidence increases exponentially as BMI decreases.^23^ At a population level, the effect of this risk factor has been predicted to be substantial owing to the widespread global prevalence of under-nutrition.^24^

A history of worm infestation has also been shown to have a significant independent association with active PTB (prevalence of 37.5% amongst cases compared with 11.1% amongst controls; adjusted OR: 11.3; *P* < 0.001). This finding is consistent with a previous case-control study conducted in northern Ethiopia, which included 230 TB cases and 510 controls. In that study, the prevalence of intestinal helminths was 71% in cases compared with 36% in controls, with an OR of 4.2 (*P* < 0.001).^25^ A case-control study conducted in south-western Ethiopia on people living with HIV and/or AIDS also showed that a history of worm infestation is significantly associated with active PTB.^26^ It is suggested that infection with helminths can lead to the development of active TB through enhancing the helper T-cell type 2 (Th-2) immune responses.^32^

Another determinant factor of active TB in the present study was exposure to a family member with known TB infection. This is also consistent with findings from a study conducted in The Gambia, where 45% of PTB cases had a family history of TB compared with 11% of the controls (OR: 7.55; *P* < 0.001). In the present study, 19% of cases reported household exposure to a known TB case, compared with 5.8% of controls (OR: 4.4; *P* = 0.008). Other studies have reported similar findings. In addition, the risk appears to increase when the contact is with a close family member as the primary TB case, compared with unrelated household members.^27,33^

Data on income were difficult to obtain, as income-generating activities were commonly carried out in the ‘informal’ economic sector. In addition, it was difficult to assess whether the income of the individual reflects the overall income of the household; however, ownership of the house remained a consistent marker of socio-economic status throughout the study and showed a statistically significant association with active PTB.

Being single was another determinant factor for development of active TB. By virtue of their marital status, single people have a different lifestyle to those who are married. This is especially the case for male patients, who often migrate to different towns in search of jobs, where they frequently live alone or with peers;^24^ therefore, in the present study, being single was independently associated with active PTB. However, further study is needed to explore the factors underlying marital status as being a determinant to active TB.

The present study also revealed that place of residence has a significant independent association with active PTB, with rural residents being at higher risk than those from urban areas. This factor has not been previously addressed by other studies and may be due to the differences in lifestyle or differences in standard of living or socio-economic status. Further study is needed to explore this association.

### Limitations

Social desirability bias in the community associated with questions about history of worm infestation can be seen as a weakness in the study design, as mild forms of infestation may have gone undiagnosed and the variable was therefore assessed based purely on personal history. In addition, selection of clinic controls is reasonable when the source population is difficult to define, but bias can be introduced during selection and the effect of the variables under study may be underestimated. Another problem is that recall bias is inherent to case-control studies. In this study, recall bias might have led to the underestimation of the effect; a short recall duration was used to limit the effect.

Another concern in this study was its failure to identify the temporal relationships between exposure factors and the result (reverse causality). This could be the situation with smoking and khat chewing, as health workers may advise newly diagnosed TB cases and their family to give up this habit.^34^ For that reason, we collected information on the duration of smoking to differentiate past from current smoking, so that reverse causality was unlikely to explain the findings.

## Conclusion

The present study highlighted many host-related and environment-related factors that contribute to the development of active PTB in humans. In this study, the most important contributing factors to developing active PTB included being single, widowed or divorced, living in rural areas, having a family member with a history of TB, smoking, being under-nourished or having a BMI of less than 18.5, being infected with HIV, and having a history of worm infestation. Furthermore, not being the owner of a house is a powerful predictor of active PTB. Measures taken to reduce the prevalence and burden of active PTB should consider these factors.

Based on our findings, the following recommendations are made to curbing TB infection and the associated burden of the disease:

- Reducing or ceasing smoking should be actively encouraged through measures such as working on long-term behavioural changes and health education.
- Efforts to stop the pandemic effect of HIV should be strengthened.
- Measures to tackle the prevalence of worm infestation in the community through different approaches should be strengthened.
- New PTB patients should be educated on how to protect their family and community. To reduce the transfer of disease, health extension workers should also provide health education regarding the care of any family members who have developed active PTB to reduce the risk of exposure in other family members.
- Further prospective cohort studies should be conducted to clearly identify other determinant factors, as the current study could not identify the temporal relationships between predictor variables and outcome variables.
